# A physical activity coaching intervention can improve and maintain physical activity and health-related outcomes in adult ambulatory hospital patients: the Healthy4U-2 randomised controlled trial

**DOI:** 10.1186/s12966-020-01063-x

**Published:** 2020-11-30

**Authors:** Stephen Barrett, Stephen Begg, Paul O’Halloran, Michael Kingsley

**Affiliations:** 1 La Trobe Rural Health School, La Trobe University, PO Box 199, Bendigo, Victoria 3552 Australia; 2 Health Promotion Department, Bendigo Health Care Group, PO Box 126, Bendigo, Victoria 3552 Australia; 3 La Trobe Rural Health School, La Trobe University, PO Box 199, Bendigo, Victoria 3552 Australia; 4 School of Psychology and Public Health, La Trobe University, Bundoora, Victoria 3068 Australia; 5 Holsworth Research Initiative, La Trobe Rural Health School, La Trobe University, PO Box 199, Bendigo, Victoria 3552 Australia; 6 Department of Exercise Sciences, University of Auckland, Newmarket, 1023 New Zealand

**Keywords:** Physical activity, Exercise motivation, Accelerometry, Public health

## Abstract

**Background:**

The Healthy 4 U-2 study sought to evaluate the effect of a twelve-week, physical activity (PA) coaching intervention for changes and maintenance in PA, anthropometrics and health-related outcomes in adults presenting to an ambulatory hospital clinic.

**Methods:**

One hundred and twenty insufficiently active adults were recruited from an ambulatory hospital clinic and randomised to an intervention group that received an education session and five 20-min telephone sessions of PA coaching, or to a control group that received the education session only. ActiGraph GT3X accelerometers were used to measure moderate-to-vigorous physical activity (MVPA) at baseline, post-intervention (3-months) and follow-up (9-months). Secondary outcome measures (anthropometrics, PA self-efficacy, and health-related quality of life) were also assessed at the three time points.

**Results:**

At baseline, the mean age and body mass index of participants were 53 ± 8 years and 31 ± 4 kg/m^2^, respectively. Relative to control, the intervention group increased objectively measured MVPA at post-intervention (*p* < 0.001) and 9 months follow-up (*p* < 0.001). At the 9-month follow-up the intervention group completed 22 min/day of MVPA (95% CI: 20 to 25 min/day), which is sufficient to meet the recommended PA guidelines. The intervention group exhibited beneficial changes in body mass (*p* < 0.001), waist circumference (*p* < 0.001), body mass index (*p* < 0.001), PA self-efficacy (*p* < 0.001), and health-related quality of life (*p* < 0.001) at the 9-month follow-up.

**Conclusions:**

This study demonstrates that a low contact PA coaching intervention results in beneficial changes in PA, anthropometrics and health-related outcomes in insufficiently active adults presenting to an ambulatory care clinic. The significant beneficial changes were measured at post-intervention and the 9-month follow-up, demonstrating a maintenance effect of the intervention.

**Trial registration:**

Prospectively registered on the Australian and New Zealand Clinical Trials Registry (ANZCTR, Trial registration number: ACTRN12619000036112.

**Supplementary Information:**

The online version contains supplementary material available at 10.1186/s12966-020-01063-x.

## Background

Insufficient physical activity (PA) is a major public health problem [1, 2], and is associated with a range of chronic diseases [3], decreased quality of life [4] and morbidity [5]. Individuals with chronic diseases are frequent users of complex hospital services [6]. This care is often delivered at ambulatory secondary care clinics through medical consultations in specialties such as general surgery, orthopaedic surgery and endocrinology. Non-emergency ambulatory services for chronic diseases account for a large proportion of healthcare expenditures [6]. Due to the increasing demands of managing chronic diseases [7], hospitals need effective and accessible prevention programs targeting high-risk individuals to increase PA and promote individual self-management [8].

Ambulatory secondary care is an important setting to target high-risk individuals to facilitate changes in PA, and may be an effective strategy for increasing PA [9]. Patients seek lifestyle advice from their health-care providers, and anticipate discussions on lifestyle choices such as PA as part of their medical care [10, 11]. Patients believe that doctors are credible sources of preventive health information, and are likely to accept doctors’ advice and instigate behaviour change [12, 13]. Surgeons can identify individuals who are likely to benefit from increasing PA, and facilitate referral pathways into behaviour change interventions as a method for increasing PA [14].

Individually tailored behaviour change interventions are effective at improving PA [15]. Key components of PA behaviour change include increasing motivation, goal setting, problem solving, social support, and performance feedback [16]. Telephone coaching is a well-recognised method of delivering PA behaviour change interventions [15]. PA telephone coaching enables a remote but personal relationship, and repeated contacts to promote behaviour change [15]. PA telephone coaching is also favourable due to its low cost, potential for wide dissemination and accessibility across geographical regions.

Research assessing the effects of telephone coaching for changes in PA has shown promise [16–19]. There is however, a lack of research investigating telephone coaching for changes in PA in the ambulatory secondary care setting. In a previous study (Healthy4U), we assessed the efficacy of telephone coaching for changes in PA in a self-selected sample of insufficiently active ambulatory secondary care patients [20]. At 3-month post-intervention, participants in the intervention group demonstrated significant improvements in PA and health-related outcomes compared to controls [20]. The present study builds upon the H4U study in a number of ways, including the use of referrals by hospital surgeons as the recruitment strategy to better integrate the PA coaching into routine secondary care. This present study also extended the follow-up measures to include a six-month post-intervention period to assess behaviour change maintenance [21]. Although PA coaching has demonstrated effectiveness in maintaining PA changes [16, 17, 22], studies that confirm long term PA behaviour change in ambulatory secondary care are scarce. The assessment of outcomes at least six-month post-intervention, where no contact with participants has been made since the intervention has ceased are required to provide a more robust measure of behaviour change maintenance in ambulatory secondary care [21].

The primary aim, therefore, was to examine the effectiveness of the PA telephone coaching intervention for change and maintenance of PA in insufficiently active secondary care patients referred by consulting hospital surgeons. Secondary aims were to investigate the effectiveness of the telephone coaching for changes and maintenance in anthropometry, PA self-efficacy, and health related quality of life in this population.

## Methods

### Design

**Fig. 1:**
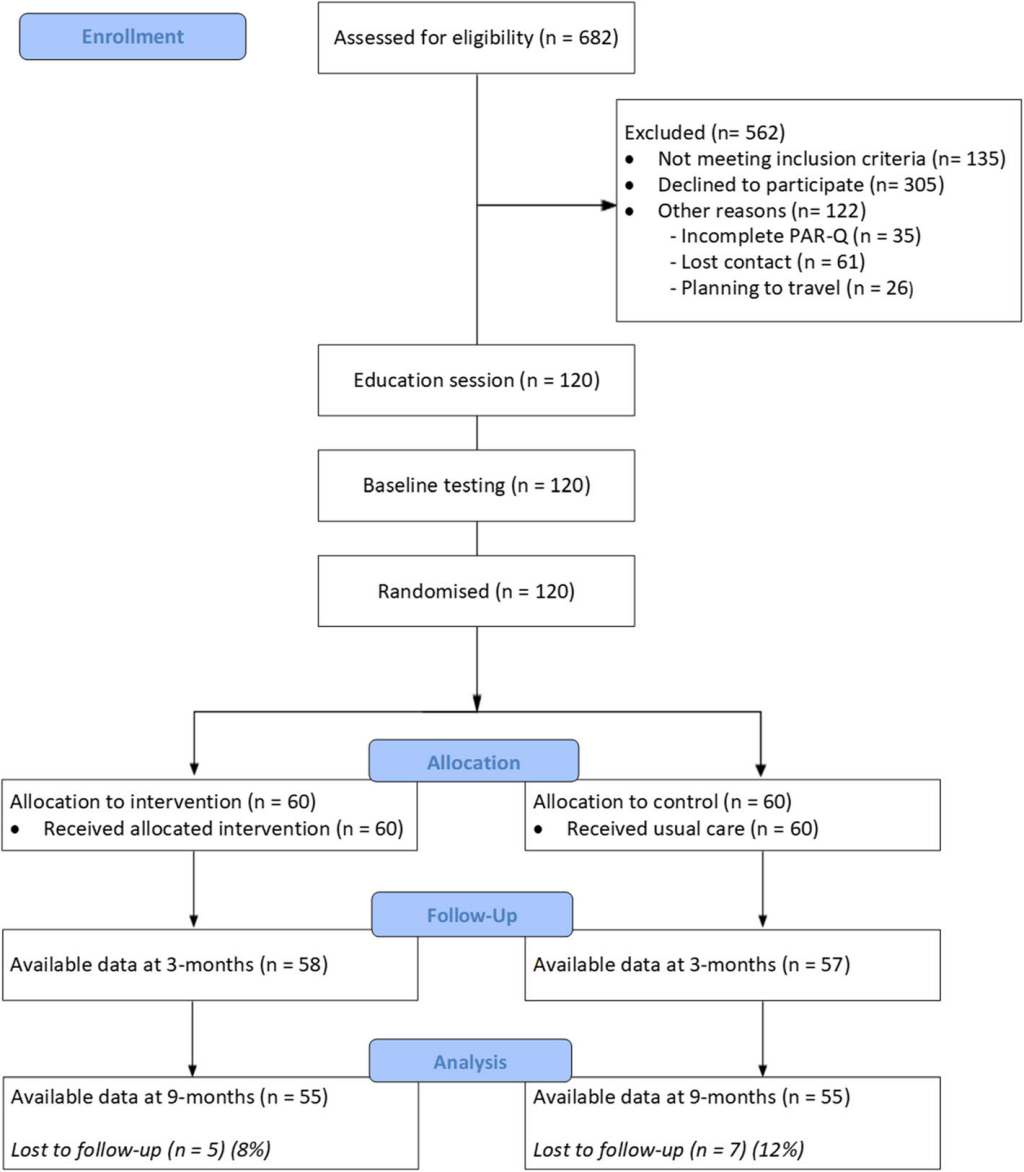
CONSORT flow diagram of the study

The study was a single-blind RCT designed and reported in line with the CONSORT statement and checklist (Fig. 1) [23], and the TIDieR checklist [24]. The completed CONSORT and TIDieR checklists are included in Additional files 1 and 2. The trial was registered with the Australian and New Zealand Clinical Trials Registry (ACTRN12619000036112) prior to participant recruitment.

**Fig. 2:**
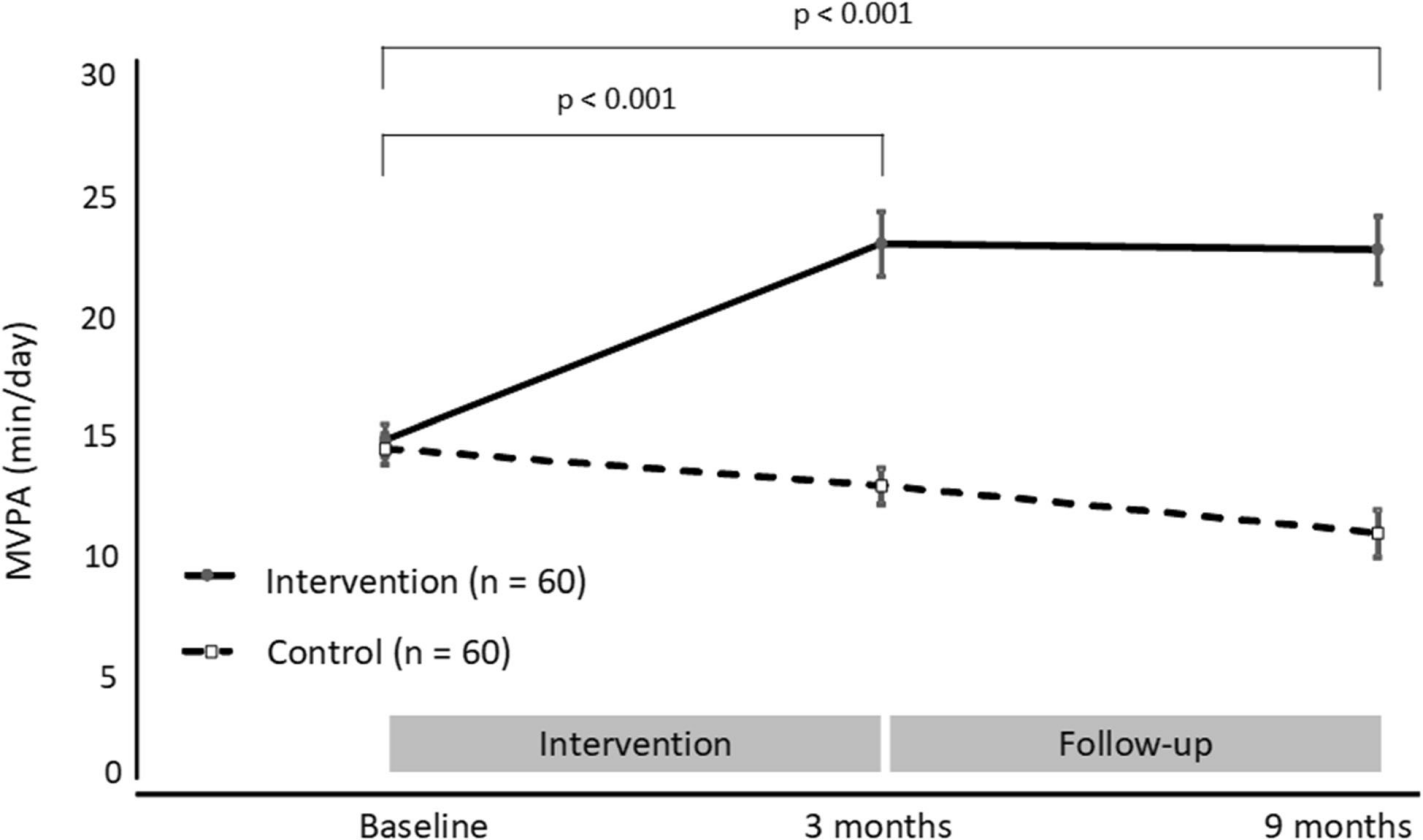
Minutes per day of moderate-to-vigorous physical activity (MVPA) for the intervention and control groups at baseline, post-intervention and follow-up

### Participants

Participants were recruited from an ambulatory, secondary care clinic in a major hospital in regional Victoria between January 2019 and September 2019. Recruitment involved consulting orthopaedic and general surgeons providing, during the normal course of the consultation, a verbal recommendation to engage in PA coaching and a sequentially numbered research flier (Additional file 3) to adult patients who, in their view, would benefit from increased PA. Potential participants contacted the research team of their own volition using information on the flyer. Sequential numbering allowed the research team to quantify both the number of individuals referred by surgeons and the number who subsequently acted on this referral.

Participants were included if they were between 18 and 69 years, and did not meet the recommended PA guidelines [25]. A single item question “As a rule, do you do at least half an hour of moderate or vigorous exercise (such as walking or a sport) on five or more days of the week?” was used to identify insufficiently physically active individuals [26]. The question has demonstrated good sensitivity (76.7%), high specificity (81.1%), and a high positive predictive value (86.7%) for identifying those not achieving the recommended 150 min of MVPA per week [26]. Exclusion criteria included: sufficiently physically active; an existing medical condition that contraindicated PA (indicated by the Physical Activity Readiness Questionnaire); deaf/hearing impaired; poor comprehension of English language; disabling neurological disorder; severe mental illness such as psychosis; learning disability; dementia and cognitive impairment; registered blind; housebound or resident in nursing home; non-ambulant; pregnancy; advanced cancer; and scheduled for surgery with 30 days of the clinic presentation.

### Procedure

Participants who were eligible and consented to take part were randomly allocated to either the intervention or the control group based using block randomization generated by a computer generated random number sequence (randomizer.org). The allocation sequence was generated by a research assistant who was not involved in the data collection process. Assignments were prepared and sealed in sequentially numbered opaque envelopes. Intervention assignment was made by opening the next envelope in the sequence, after: (1) the recruiter had determined eligibility for the study; (2) participants had consented to take part; (3) attendance at an education session was confirmed; and (4) baseline measurements were completed.

### Intervention

All enrolled participants attended an education session prior to group allocation. The education session was a facilitated learning session based around PA self-management and was carried out using a self-determination theory framework [27]. Self-determination theory is a motivational theory commonly used to elicit changes in PA [28]. Self-determination theory was used in this group setting to support, educate and motivate participants around PA changes [27].

The intervention group completed a telephone-based coaching intervention that comprised integrated motivational interviewing and cognitive behaviour therapy (MI-CBT). The coaching was delivered in five by 20-min sessions over 12 weeks. The intervention schedule, theories and techniques are displayed in Table 1. The intervention was delivered using a motivational interviewing (MI) framework, with MI used as the underpinning approach to influence motivation, ambivalence and self-efficacy to be physically active [29]. Motivational interviewing microskills (open-ended questions, affirmations, reflections and summaries) were utilised in all sessions [29]. The cognitive behaviour therapy (CBT) coaching focused on six theory-derived determinants of PA: PA outcome expectations, PA outcome experiences, PA values, PA barrier self-efficacy, social support and relapse prevention [30]. The (CBT) strategies were incorporated within a MI framework to support PA change and maintenance [31]. The intervention was delivered by an experienced Australian Health Practitioner Regulating Authority registered physiotherapist. The clinician was trained in MI-CBT, including workshop attendances, and one-on-one coaching from an experienced practicing psychologist. The clinician previously delivered 145 h of MI-CBT in the H4U study [20]. Fidelity was assessed using the MI-CBT fidelity scale [32] using audio-recorded sessions.

**Table 1:** Physical activity coaching schedule, content, theory, determinants and behaviour change techniques

Session	Week	Session determinants	Content	Techniques
1	1	Physical activity expectations; Physical activity past experiences; Physical activity self-efficacy; Physical activity values.	• Exploration of current and historical physical activity behaviours; • Identify telephone coaching outcome expectations; • Identify physical activity outcome expectations; • Determine level of motivation for increasing physical activity (e.g. how motivated are you to increase physical activity on a scale of 1–10? Why did you give it a 3, as opposed to a 4 or 5?); • Identify and address unrealistic physical activity expectations; • Assess barriers to physical activity; • Discuss goals and action plans.	Motivational interviewing strategies: • Open ended questions; • Affirmations; • Reflections; • Summaries; • Develop discrepancy; and • Illicit change talk. Cognitive-behavioural techniques: • Elicit PA outcome expectations and experiences; • Elicit values and physical activity priorities; • Identify physical activity barriers and problem solving; • Goal setting –behavioural; • Action planning.
2	2	Physical activity outcome expectations; Experience regarding goal setting.	• Review of goal progress from session 1; • Barrier identification and determine level of self-efficacy for overcoming barriers (e.g. how confident are you to overcome barrier X on a scale of 1–10? Why did you give it a 3, as opposed to a 4 or 5?); • Progress and amend action-plan and goals; • If physical activity goals involve program based activities (e.g. strength training, walking groups) individual to source contact details.	Motivational interviewing strategies as above. • Illicit and explore change talk. Cognitive-behavioural techniques: • Problem solving; • Goal setting; • Focus on past success; • Prompt experiential learning through trial and error.
3	4	Outcome expectations and experiences in relation to physical activity goal progress.	• Review of goals and progress from session 2; • Explore current experiences of physical activity; • Barrier identification and self-efficacy strategies for overcoming barriers; • Discuss self-monitoring strategies to monitor goal (e.g. physical activity tracking); • Discuss intervention timelines and action plan for the next two weeks.	Motivational interviewing strategies as above. Cognitive-behavioural techniques: • Review of physical activity behaviour and outcome goal(s); • Elicit current physical activity outcome experiences; • Goal planning, and what-then plans; • Education regarding self-monitoring of behaviour or outcomes; • Relapse prevention.
4	6	Physical activity outcome expectations; Exercise self-efficacy; Coping strategies; Future planning.	• Review of progress from session 3; • Explore current experiences of physical activity; • Relapse prevention - tailored to individual needs; • Discuss intervention timelines and action plan for the next six weeks.	Motivational interviewing strategies as above. Cognitive-behavioural techniques • Elicit current physical activity outcome experiences; • Coping strategies (e.g. physical activity pacing, planning); • Engaging social support; • Relapse prevention
5	12	Theory of behavioural maintenance; Relapse prevention;	• Intervention recap; • Review of progress from previous session and intervention as a whole; • Identify what has helped PA changes; • Identify what can helped PA maintenance; • Relapse prevention – identification of potential future scenarios, and what-then plans for overcoming issues (e.g. if I experience X, then I will do Y); • Additional follow-on services – community health promotion services/exercise services.	Motivational interviewing strategies as outlined above. Cognitive-behavioural techniques: • Action planning - focus on past and current success; • Problem solving – what-if planning. • Relapse prevention.

### Outcome measures

Participants’ outcome measures were recorded at baseline, after 3 months of intervention (post-intervention) and at 9 months (follow-up) by assessors blinded to the study group assignment. Information on treatment allocation was not provided to data collectors. The data collection forms only contained unique identifier codes assigned to each participant.

#### Primary outcome

The primary outcome of interest was the change in MVPA (minutes/day) over time. MVPA was objectively assessed using a tri-axial accelerometer (wGT3X-BT; Actigraph, USA). Participants were instructed to wear the accelerometer on their hip at all times over 7 consecutive days, excluding sleep and water-based activities. PA was calculated using the manufacturers software (Actilife; Actigraph, USA) with cut points by Freedson Adult (1998) used to provide daily measures of MVPA (> 1951 cpm) [33]. Accelerometer wear time was based on activity counts per minute (CPM). Non-wear time was defined as 60 min or more of consecutive activity counts of zero, with a spike tolerance of 2 min and 100 cpm. Participants used logbooks to report activities and periods of accelerometer non-wear. Non-wear time was compared to participants’ notes on their logbook. A minimum of 10 h per day was used as the cut-off for a valid day of measurement and a minimum of 5 days of data were required including at least 1 weekend day [34]. Weekly MVPA was computed based on the average of all valid days per person. A daily average of 21 mins of MVPA over a 7 day period is sufficient to meet the 150 min of MVPA recommended by the Australian PA guidelines [25].

#### Secondary outcomes

Change over time for the following secondary outcomes was also assessed. Waist circumference was measured to the nearest 0.1 cm using a rigid anthropometric measuring tape (Lufkin, US). Body mass was recorded to the nearest 0.1 kg using a calibrated scale (model 813; Seca, Germany). Free standing stature was recorded to the nearest 0.1 cm using a calibrated equipment with the participant barefoot (Portable stadiometer; Seca, Germany). Body mass index (BMI) was calculated by dividing body mass by the square of height. Self-efficacy to be physically active was measured using a validated PA self-efficacy survey [35]. Health-related quality of life (HrQoL) was measured using the Medical Outcomes Study Short Form 12 Health Survey (SF-12) which is a reliable tool with published psychometric support [36].

### Study size

Utilising data from the H4U study [20], a sample size of 50 participants per arm was calculated to be sufficient to detect a between group difference of 30 ± 13 (mean ± SD) mins/week MVPA, with the alpha set at 0.05, and the power set at 0.80. Protecting against a drop-out rate of 20% over the 9-month study duration, 60 participants were recruited and randomised into each arm.

### Data analyses

Analyses were carried out using IBM SPSS Statistics for Windows (Version 26.0; IBM Corp., USA) and statistical significance was set at an alpha of 0.05. Data were assessed for normal distribution by Shapiro-Wilk tests. Homogeneity of variances and covariances were assessed by Levene’s test and Box’s M test, respectively. Grouped data are presented as mean ± standard deviation. For the main analyses, a series of mixed-model ANOVAs (within: time; between: intervention) were used to assess the effects of the PA coaching intervention on each of the outcome variables separately. Mauchly’s test was consulted and Greenhouse–Geisser correction was applied if the assumption of sphericity was violated. A significant interaction effect was interpreted to demonstrate that the change in dependent variables was influenced by the intervention. Where statistically significant interactions were observed simple main effects analyses were carried out. Where data were in breach of Shapiro-Wilks test of normality, sensitivity analyses were performed. Data were explored for significant outliers and repeat sensitivity analyses were undertaken on data with outliers removed. Repeated sensitivity analyses provided no indication that the outliers had a significant effect on the outcome; therefore, all data were included in analyses. Intention-to-treat analysis was used for missing data using the last observation carried forward approach [37]. Repeat sensitivity analyses were undertaken on data with and without imputing the last-observation-carried forward value and provided no indication that the imputed values had a significant effect on the outcome.

## Results

Utilising the sequentially numbered study fliers, we know that 2076 individuals were provided with the study information and a recommendation to engage with the PA coaching service. The H4U-2 study team were contacted by 682 individuals (33%), of which 120 consented and were eligible to participate in the study (Fig. 1). Of the 305 individuals who chose not to participate, 282 did not want to enroll in the study but did want support to be more physically active; these individuals proceeded to enroll in the hospital’s standard PA health promotion program. These data indicate that when ambulatory hospital patients are recommended by a surgeon to participate in a PA coaching intervention, 33% demonstrate sufficient interest to enquire about the service, and almost 20% will take up a PA coaching intervention.

One hundred and twenty participants (68% female) completed baseline assessment and were subsequently randomised into the intervention or control groups. Baseline demographic and clinical characteristics between the intervention and control groups were similar (Table 2). Drop-out rates were low with 115 participants completing their 3-month assessment, and 108 participants completed their 9-month assessment (Fig. 1). Mean accelerometer wear time was 14 ± 3 h per day and 6.1 ± 0.8 days per week (out of 7 days per week). Almost all participants (96%) enrolled into the intervention arm received their scheduled 5 sessions of PA telephone coaching. The mean duration of each intervention session was 18 ± 4 min. Results from the assessment of intervention fidelity revealed that the intervention provider demonstrated competence during the delivery of MI-CBT structure and core skills.

**Table 2:** Characteristics of participants at baseline

Variable	Total	Intervention	Control
	120	60	60
**Age (years)**	53 ± 8	54 ± 8	53 ± 7
**Sex: female, n (%)**	81 (68%)	40 (67%)	41 (68%)
**Stature (cm)**	166 ± 8	165 ± 9	167 ± 7
**Weight (kg)**	84.4 ± 9.4	84.5 ± 9.9	84.3 ± 9.1
**BMI (kg/m**^**2**^**)**	30.5 ± 4.3	31.0 ± 4.4	30.0 ± 4.2
**MVPA (min/day)**	14.5 ± 4.9	14.7 ± 5.2	14.3 ± 4.7
**PA Self-efficacy**	25 ± 4	24 ± 4	25 ± 4
**Smoker, n (%)**	12 (10%)	7 (10%)	5 (10%)
**Obesity, n (%)**	62 (52%)	30 (50%)	32 (53%)
**Hypertension, n (%)**	38 (32%)	20 (33%)	18 (30%)
**OA/RA, n (%)**	42 (35%)	22 (37%)	20 (33%)
**Depression/anxiety, n (%)**	25 (21%)	12 (20%)	13 (22%)
**Employment status, n (%)**			
**Full time**	48 (40%)	25 (42%)	21 (35%)
**Part time**	39 (32%)	19 (32%)	22 (37%)
**Unemployed**	11 (10%)	5 (8%)	8 (13%)
**Retired**	22 (18%)	11 (18%)	9 (15%)
**Education, n (%)**			
**Year 10/11**	3 (3%)	1 (2%)	2 (3%)
**Year 12**	31 (26%)	13 (22%)	17 (28%)
**Cert I-IV**	42 (35%)	24 (40%)	19 (32%)
**Diploma**	26 (21%)	14 (23%)	12 (20%)
**Bachelor or higher**	18 (15%)	8 (13%)	10 (17%)

Group data expressed as means ± standard deviations. Figures in parentheses are proportions. *BMI* Body mass index; *MVPA* Moderate-to-vigorous physical activity; *OA* Osteoarthritis; *RA* Rheumatoid arthritis

Repeated measures ANOVAs demonstrated a significant group by time interaction for daily MVPA, indicating that changes from baseline differed between the intervention and control group (*p* < 0.001; Fig. 2). The intervention group significantly increased MVPA over time, undertaking 22 min/day (95%CI: 20 to 25 min/day) at 9-month follow-up. In contrast, the control group significantly decreased MVPA over the same time, undertaking 10 min/day (95%CI: 8 to 13 min/day) at follow-up. This translates to a between group difference for MVPA of 12 min/day (95%CI: 9 to 15 min/day) at 9 months.

Statistically significant group by time interaction effects were found for all secondary outcomes (Table 3). For the intervention group, at follow-up there were significant changes body mass (− 2.1 kg, 95%CI: − 1.6 to − 2.7 kg), waist circumference (− 1.3 cm, 95%CI: − 0.9 to − 1.7 cm), and BMI (− 0.8 kg/m2, 95%CI: − 0.6 to − 1.0 kg/m2). Relative to the control group, the intervention group also demonstrated significant changes in PA self-efficacy (6 points, 95%CI: 4 to 8 points) and HrQoL (0.02 units, 95%CI: 0.002 to 0.04 units).

**Table 3:** Means and standard deviations for outcome measures at 3 months and 9 month follow-up

Outcome	Intervention			Control			Analyses	
	Baseline	Post-Intervention	Follow-up	Baseline	Post-Intervention	Follow-up	Time x Group (F)^**a**^	Effect size^**b**^
MVPA (min/day)	15 ± 5	23 ± 10	22 ± 10	14 ± 5	13 ± 6	10 ± 6	28.7*	0.20
Waist circumference (cm)	97.6 ± 11.7	97.6 ± 11.7	96.3 ± 11.4	97.4 ± 11.4	97.8 ± 11.1	98.2 ± 11.1	45.9*	0.28
Body mass (kg)	84.5 ± 9.9	83.2 ± 9.6	82.4 ± 9.4	84.3 ± 9.1	85.3 ± 8.9	85.8 ± 8.8	107.8*	0.48
BMI (kg/m^2^)	31.0 ± 4.5	30.6 ± 4.4	30.2 ± 4.3	30.0 ± 4.2	30.4 ± 4.1	30.5 ± 4.2	108.2*	0.49
PA self-efficacy (Scale)	24 ± 4	28 ± 6	30 ± 6	25 ± 4	25 ± 4	22 ± 4	53.3*	0.31
HrQoL (Scale)	0.63 ± 0.06	0.64 ± 0.06	0.65 ± 0.07	0.63 ± 0.07	0.63 ± 0.07	0.61 ± 0.06	8.7*	0.07

Group data are means ± standard deviations. MVPA: moderate-to-vigorous physical activity; *BMI* Body mass index; *HrQoL* Health-related quality of life. *p < 0.05. ^a^ interaction effect of time by group on dependent variable; ^b^ Partial eta-squared

The proportion of individuals undertaking sufficient daily MVPA to meet the PA guidelines was similar across both groups at baseline, with 8% (*n* = 5) of the intervention group and 13% (*n* = 8) of control group sufficiently active. In the control group this proportion decreased to 10% (*n* = 6) at 3 months, and decreased further to 3% (*n* = 2) at 9 months. In contrast, the proportion of individuals in the intervention group undertaking sufficient daily MVPA increased to 55% (*n* = 33) at 3 months, and dropped slightly to 52% (*n* = 31) at 9-months.

## Discussion

Physical activity telephone coaching resulted in a significant increase in MVPA that was maintained at 9-months in adults attending ambulatory secondary care clinics. The intervention also resulted in significant improvements in body mass, waist circumference, BMI, PA self-efficacy, and HrQoL. The positive changes exhibited in outcomes at 3-months and 9-months indicate short-term and maintenance effects of the intervention. The attrition rate of 10% was low. This study offers important information on the potential effects that can be achieved by a targeted, patient-centred PA lifestyle intervention delivered in an ambulatory hospital setting.

The intervention group significantly increased MVPA at 3 months (post-intervention) and maintained this change at 9-month follow-up. In contrast, the objectively assessed MVPA of the control group declined below baseline at 3 months, and declined further at follow-up. Very few studies have analysed the long-term effects of remotely delivered PA coaching [15, 38]. The effect size observed in this study (0.20) was higher than that found (0.11) in a meta-analysis of remote PA interventions for self-reported PA change [39]. Additionally, following a PA coaching intervention, self-reported PA was maintained after a no-contact follow-up, whereas objectively assessed PA decreased [40]. Objectively measured increases in MVPA in the intervention group that were maintained following a 6-month no-contact period indicate that change in behaviour was maintained at 9 months.

The majority of intervention participants undertook sufficient PA to meet the PA guidelines at 3 months and 9 months. This was in stark contrast to the control group where less than 10% of participants were classified as sufficiently active at 3 months and 9 months. The increase in MVPA in the intervention group and the group differences between the control and the intervention group are highly relevant. Previous studies have documented that 15 min a day of MVPA can decrease chronic disease risk [3], attenuate the risk of sedentary behaviours [41], and reduce all-cause mortality [42].

Compared to control, the intervention participants experienced improvements in their body mass, waist circumference and BMI from baseline to follow-up. The improvements in the intervention group were maintained during the no contact period from the end of coaching sessions for a further 6 months. The magnitude of long-term change in anthropometrics is comparable to changes reported in other studies using telephone coaching interventions, though none of these were conducted in the ambulatory secondary care setting [22, 43]. The positive changes in the intervention group and the group differences between the intervention and control group are relevant, and could have important population-health implications for addressing chronic disease risk factors. Even at modest levels, weight loss and decreases in waist circumference are beneficial for chronic disease risk reduction [44, 45]. The recruitment into this study was based upon changing PA, not anthropometrics. Additionally, intervention components only addressed issues relating to PA beliefs, attitudes and plans. The positive results for these secondary outcomes indicates that PA moderately, but significantly induces anthropometric changes, and it is important in the maintenance of these changes [46].

Baseline scores for PA self-efficacy indicated that both groups had low-to-moderate confidence in their ability to be physically active. In the control group PA self-efficacy decreased significantly over time. This contrasted with the trajectory of the intervention group, who increased PA self-efficacy at 3 months, and even further at 9 months. The changes in PA self-efficacy potentially mediated the changes in PA amongst the groups [47]. The intervention group increased PA self-efficacy and PA, while the control group demonstrated simultaneous decreases in both these outcomes. The MI-CBT intervention demonstrated efficacy in improving psychological determinants in the short and long term, including self-efficacy to overcome exercise barriers and maintain change. Self-monitoring, goal setting, feedback on outcome of behaviour and action planning are known to be effective for behaviour change, and can strengthen autonomy which is important for maintenance of change [48]. Integrated MI-CBT strategies can influence the determinants associated with PA maintenance if implemented correctly, and appear to be particularly important for long-term effects [49].

### Strengths and limitations

Using consulting hospital surgeons to identify insufficiently physically active individuals was an important strength of this study. Once identified, surgeons discussed the need for PA change with insufficiently active patients and referred them to the study. This approach was based on hospital surgeons’ stated preferences, previously ascertained, for clear referral pathways into specialist behaviour change programs [14], and demonstrates how preventive health can be successfully embedded into routine ambulatory hospital care. The use of sequentially numbered study fliers permitted the calculation of the PA intervention interest and uptake. It is encouraging in this respect that one-third of the individuals made contact with the study team after referral by a hospital surgeon, and almost 20% went on to undertake PA coaching of some type.

The uptake of the intervention itself is also promising given the opt-in procedure that was used and the eligibility criteria that were applied. Many insufficiently active people are not ready to change important behaviours and are therefore unlikely to volunteer for a study such as this [50]. Nevertheless, the individuals who did enroll in the study still needed to make those changes and we were able to demonstrate effectiveness in this group due to the robust nature of the RCT study design. This strengthens confidence in the transferability and scalability of our findings. In the H4U study we demonstrated the efficacy of PA telephone coaching in self-selected sample of ambulatory care [20]; in this study we have demonstrated its effectiveness when used as the end point in a referral pathway starting with consultant surgeons working in ambulatory secondary care.

The participant retention rate in this study was high, with only 12 participants lost to follow-up. Intervention adherence rate was also high, with 96% of participants receiving all 5 sessions of telephone coaching. The use of objectively measured PA at all time points was a considerable strength of the study, offering precise estimates of PA intensity. For a regional hospital, delivering the PA intervention via telephone permitted extending the reach to both geographically and socially disadvantaged areas. A limitation of the study may be the involvement of only one hospital, though this permitted the continuation of a previous body of work towards integrating preventive health in that hospital. Additionally, the broad generalizability of these findings might be difficult because the majority of participants were female, and had a BMI that classified them in the category of obese.

## Conclusions

Ambulatory hospital appointments provide an important opportunity for initiating PA behaviour change. The H4U-2 trial demonstrates that PA telephone coaching is an efficacious tool for the promotion of a physically active lifestyle in adults and facilitates sustained behaviour change maintenance 6 months post-intervention. Physical activity coaching also resulted in significant sustained improvements in anthropometrics, PA self-efficacy and HrQoL. Telephone coaching interventions in ambulatory hospital care contribute to substantial improvements in participant’s health outcomes, and are effective for the prevention and management of chronic disease.

## Supplementary Information

**Additional file 1.**

**Additional file 2.**

**Additional file 3.**

## Data Availability

The dataset used and analysed during the current study are available from the corresponding author on reasonable request.

## Abbreviations

ANOVA: Analysis of Variance
BMI: Body Mass Index
CBT: Cognitive Behaviour Therapy
CI: Confidence Interval
HrQoL: Health- Related Quality of Life
MI: Motivational Interviewing
MVPA: Moderate-to-Vigorous Physical Activity
MI-CBT: Integrated MI and CBT
PA: Physical Activity
RCT: Randomised Controlled Trial
SD: Standard Deviation
SDT: Self-Determination Theory
