# Supplementary figures and images for: A physical activity coaching intervention can improve and maintain physical activity and health-related outcomes in adult ambulatory hospital patients: the Healthy4U-2 randomised controlled trial
Source: Int J Behav Nutr Phys Act. 2020 Nov 30;17:156. doi: 10.1186/s12966-020-01063-x (PMC7708221; doi:10.1186/s12966-020-01063-x)

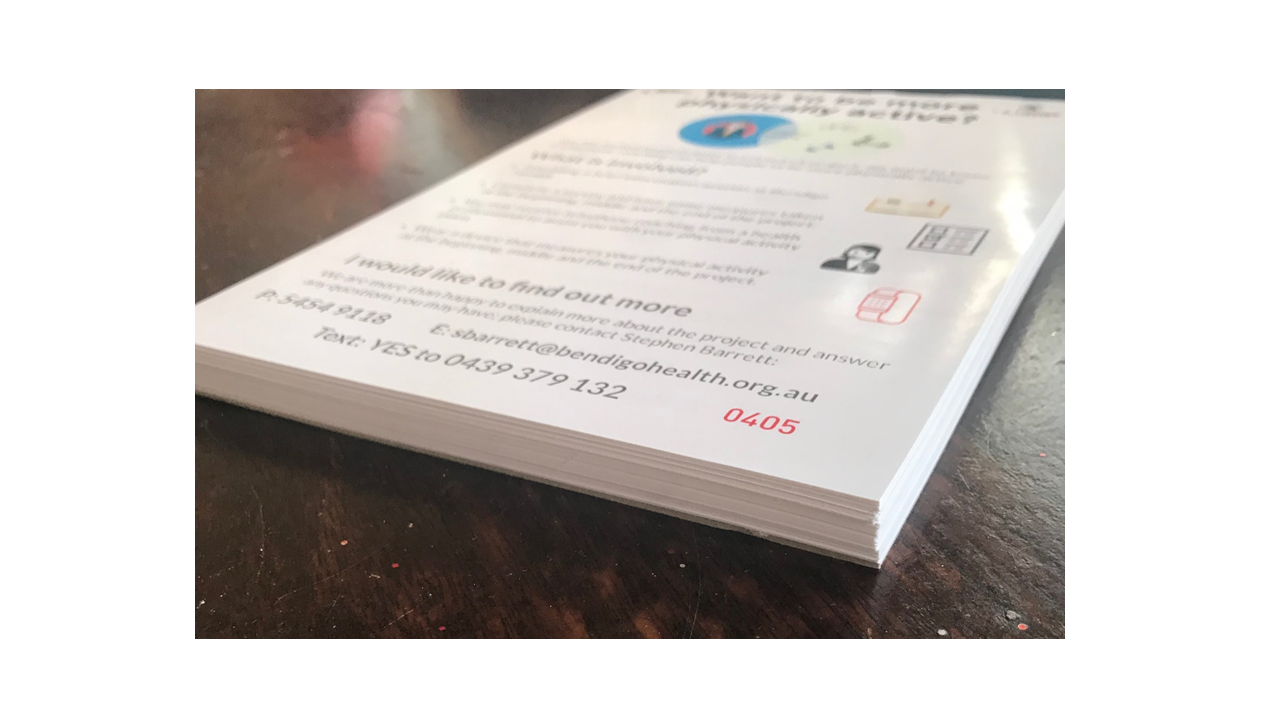

Supplement: Supplementary file 3 — Additional file 3. [file 12966_2020_1063_MOESM3_ESM.png]
